# Increased Cumulative Incidence of Dermatomyositis in Ulcerative Colitis: a Nationwide Cohort Study

**DOI:** 10.1038/srep28175

**Published:** 2016-06-21

**Authors:** Chia-Chun Tseng, Shun-Jen Chang, Wei-Ting Liao, Ya-Ting Chan, Wen-Chan Tsai, Tsan-Teng Ou, Cheng-Chin Wu, Wan-Yu Sung, Ming-Chia Hsieh, Jeng-Hsien Yen

**Affiliations:** 1 Graduate Institute of Clinical Medicine, College of Medicine, Kaohsiung Medical University, Kaohsiung, Taiwan; 2 Department of Internal Medicine, Kaohsiung Municipal Ta-Tung Hospital, Kaohsiung, Taiwan; 3 Department of Kinesiology, Health and Leisure Studies, National University of Kaohsiung, Kaohsiung, Taiwan; 4 Department of Biotechnology, College of Life Science, Kaohsiung Medical University, Kaohsiung, Taiwan; 5 Division of Rheumatology, Department of Internal Medicine, Kaohsiung Medical University Hospital, Kaohsiung, Taiwan; 6 Graduate Institute of Medicine, College of Medicine, Kaohsiung Medical University, Kaohsiung, Taiwan; 7 Division of Endocrinology and Metabolism, Department of Internal Medicine, Changhua Christian Hospital, Changhua, Taiwan; 8 Graduate Institute of Integrated Medicine, China Medical University, Taichung, Taiwan; 9 Institute of Biomedical Sciences, National Sun Yat-sen University, Kaohsiung, Taiwan

## Abstract

On a molecular level, two autoimmune diseases: ulcerative colitis (UC) and dermatomyositis share common genetic determinants. On a clinical level, case reports evidenced the co-occurrence of these two diseases. We therefore hypothesize that UC is potentially associated with increased cumulative incidence of dermatomyositis. The goals of this retrospective cohort study were to evaluate whether UC is associated with increased cumulative incidence of dermatomyositis independent of sex and age. For comparison, we also assessed the cumulative incidence of polymyositis in UC and control subjects. The study enrolled 3,133 UC subjects and 14,726 control subjects. The cumulative incidence of dermatomyositis was significantly higher in UC than that of control subjects (p = 0.026), but the cumulative incidence of polymyositis was comparable between UC and control subjects (p = 0.596). UC was independently associated with the increased incident dermatomyositis (hazard ratio: 6.19, 95% confidence interval = 1.77–21.59, p = 0.004) after adjusting for sex, age, and concomitant rheumatoid arthritis, systemic lupus erythematosus, and systemic sclerosis. Similar trends of increased dermatomyositis in UC were observed when patients were stratified based on sex and age. In conclusion, our findings suggest that UC is probably associated with increased cumulative incidence of dermatomyositis, independent of sex, age, and concomitant autoimmune diseases.

Both the incidence and prevalence of ulcerative colitis (UC) is increasing worldwide, affecting approximately 0.5% of the general population in the Western world^1^. Epidemiological data, genetic evidence and clinical features suggest that UC is a polygenic disease. Despite advances in medical management, morbidity remains high, with 30% of affected individuals undergoing colectomy^2^ due to failure of medical therapy, among other reasons. Though the precise etiology is not completely understood, the current hypothesis is a dysregulated mucosal immune response to commensal gut flora or microbiota in genetically susceptible individuals. Recent meta-analyses have identified several susceptibility loci associated with UC, including *vitamin D receptor (VDR)*^3^ and *interferon regulatory factor 5 (IRF5)*^4^. These two susceptibility genes are also associated with dermatomyositis (DM)^5^,^6^, another rarer autoimmune disease compared to UC that affects the musculoskeletal system and respiratory tract. Interestingly, there are several case reports describing the overlapping of these two autoimmune diseases^7^,^8^. The low occurrence of either UC or DM has made accumulating sufficient cases to analyze cumulative incidence and characteristics of each challenging. A high cumulative incidence of cases with both UC and DM co-occurring would suggest genetic overlapping of the two diseases. Studies have shown that, the association of UC with other autoimmune diseases is an important factor in the prognosis of patients diagnosed with UC^9^. Additionally, early recognition of patients with concurrent DM is associated with higher remission rates, shorter treatment period, and better outcomes for patients diagnosed with DM^10^. We thus performed a nationwide cohort study to evaluate the cumulative incidence of DM in UC patients and examined the association between these two diseases. Because there are data indicating that DM and polymyositis (PM) share common pathological and immunological characteristics^11^,^12^, we also utilized this nationwide cohort study to estimate the cumulative incidence of PM in UC patients.

## Methods

This nationwide cohort study was established using data from the National Health Insurance Research Database (NHIRD) and data from the Registry of Catastrophic Illness. Because these two databases were released with identification numbers encrypted, the researchers were blinded to the identity of individuals. Thus the analysis of the databases was exempt from the ethical approval by the Institutional Review Board of Kaohsiung Medical University Hospital (KMUHIRB-EXEMPT(I)-20150051).

### Source of Sample Subjects

Taiwan initiated its National Health Insurance (NHI) program in 1995 to provide affordable health care for all citizens, with coverage rate more than 99.6%^13^. The NHI medical claims database, managed by Taiwan’s National Health Research Institutes (NHRI), has collected the data of all ambulatory care and in-patient claims from NHI program enrollees. The NHI database diagnosis coding mirrors the International Classification of Diseases, Ninth Revision (ICD-9), Clinical Modification diagnostic criteria. The NHRI offered NHIRD 1,000,000 random subjects for this study, which comprised registration data on 1,000,000 beneficiaries randomly sampled from the original NHIRD. This was a representative national sample, with comparable distribution in sex, age, or average insured payroll-related amount between the claimants in the sampled data and the original NHIRD^14^. Every record in the NHIRD contains information about the date of doctor visit, age, sex, and diagnosis of patients. We used a longitudinal cohort of 1,000,000 subjects from January 1, 1998, to December 31, 2011.

Another database offered by NHRI is the Registry of Catastrophic Illness. Every record in the Registry of Catastrophic Illness contains information about the date on which the patient received their first diagnosis of catastrophic illness, the ICD-9 of catastrophic illness, the sex and age of the patient. To avoid financial hardship for families coping with major illnesses, the NHI specifies 31 categories of catastrophic illness (including UC, DM, and PM) for which patients are waived from any copayment. To be registered in the Registry of Catastrophic Illness for UC, the attending physician of these patients, usually a gastroenterologist, must submit related information, including the clinical history, endoscopic, and/or histological findings. Every case must be validated by another expert gastroenterologist to be formally approved and registered. Application of catastrophic illness certificate for DM or PM requires a thorough clinical and laboratory survey that fulfills the criteria proposed by Bohan and Peter^15^,^16^. After chart review, particularly reviewing clinical symptoms of muscle weakness, dermatological manifestations, electromyography results, muscular biopsy results, and levels of muscle enzymes (e.g. creatine kinase), lactate dehydrogenase, aspartate aminotransferase and alanine aminotransferase, only those with the diagnosis of DM or PM are issued a catastrophic illness certificate for the diseases.

To ensure coding accuracy, the NHI Bureau routinely implements cross-checks of each hospital claim with medical charts. Punitive measures were taken against coding infractions to avoid diagnosis up-coding. However, NHI’s reimbursement system ties a hospital’s reimbursement to its patient severity level. As a result, it is in the best interest of a hospital to accurately code diagnoses and care items. These databases have also been utilized for epidemiologic studies of various autoimmune diseases in numerous articles, including UC, DM, and PM^17^,^18^,^19^,^20^. Therefore, the high standard set by the databases allows researchers to analyze the epidemiological profile using the data.

### Cohort Selection

Patients with newly diagnosed UC (ICD-9 code: 556)^17^ from the Registry of Catastrophic Illness for January 1, 1998, to December 31, 2011 were identified as the UC cohort. We only included patients older than 20 years with no diagnosis of DM (ICD-9: 7103) or PM (ICD-9: 7104) before or within 1 year after their diagnosis of UC. Previous studies^17^,^18^,^19^ also utilized ICD-9 to identify patients with UC, DM, and PM in these databases. We further assigned the date on which they received a diagnosis of UC in the Registry of Catastrophic Illness as the entry date for UC patients. For every UC patient, we randomly selected at most five control subjects without UC, matched in terms of sex, age, and entry date. Control subjects could have other diseases except UC, such as rheumatoid arthritis (RA), systemic lupus erythematosus (SLE), and so on. We recruited control subjects from NHIRD. The entry date for UC patients was the date on which the patients received their diagnosis of UC in the Registry of Catastrophic Illness. Similarly for control subjects, the entry date was a matched date on which control subjects used any medical services. Moreover, we ensured that none of our control subjects had ever received a diagnosis of DM or PM prior to or within one year of their entry date. We used this process to ensure that both cohorts shared the same baseline follow-up time. Patients who were followed less than 1 year were excluded from the analysis due to inadequate duration to assess long-term exposure status^21^.

### Outcome Definition

Any participant with a DM (ICD-9: 7103) or PM (ICD-9: 7104) diagnosis was identified from the Registry of Catastrophic Illness^18^,^19^. The endpoint of follow-up was: the date of last visit before December 31, 2011, or the diagnosis date of DM or PM in the Registry of Catastrophic Illness, whichever came first.

### Assessment of Covariates

Because other autoimmune diseases including: RA, SLE, and systemic sclerosis (SSc)^22^, might be associated with DM or PM, we also ascertained the presence of RA (ICD-9: 7140), SLE (ICD-9: 7100), and SSc (ICD-9: 7101) from the Registry of Catastrophic Illness. Previous studies^18^,^19^ also utilized ICD-9 to identify patients with RA, SLE, and SSc in this database.

### Statistical Analysis

Since patients with DM or PM diagnosed before or within 1 year after the diagnosis of UC were excluded from the study, the first year after an UC diagnosis was not included in the follow-up time. The follow-up time was therefore defined as starting one year after UC diagnosis for all subjects. The follow-up period was calculated from a year after the cohort entry date to the end of follow-up.

We first evaluated the incidence rate of DM/PM and the corresponding 95% confidence interval (95% CI) in patients with UC and control subjects with person-years as the denominator under the Poisson assumption using Exact method^23^. We then used the Kaplan-Meier analysis to estimate the cumulative incidence of DM or PM after enrollment and differences in the full time-to-event distributions between different groups were compared by a log-rank test. After confirming the assumption of proportional hazards by Schoenfeld residuals trend tests, which examined the interactions between predictors and event time, we constructed Cox proportional hazard regression models with age, sex, RA, SLE, SSc, and UC adjusted simultaneously in the model. Statistical significance was inferred at a two-sided *p* < 0.05. All analyses were performed with the SPSS (v19.3) after mining the national medical records using the PERL (v5.8).

## Results

### Patient Characteristics

This study included a total of 3,133 patients with UC and 14,726 control subjects for the period spanning 1998 through 2011. The mean age was 50.42 ± 16.52 in male UC subjects and 49.70 ± 16.00 in male control subjects, 53.76 ± 15.38 in female UC subjects and 53.02 ± 14.82 in female control subjects (Table 1). The distribution by age was comparable between UC patients and control subjects in both male (p = 0.298) and female (p = 0.244) groups.

### DM and PM Incidence Rate in the UC Cohort

A total of 12 cases of incidental DM were identified during the follow-up period, 5 in the UC group and 7 in the control subjects. The incidence rate of DM was 0.36 cases per 1000 person-years (95% CI = 0.12–0.85) in the UC cohort, compared to 0.11 cases per 1000 person-years (95% CI = 0.04–0.22) in the control group (Table 2). UC tended to have higher incidence rate of DM than control subjects. Because age modified risk of extraintestinal manifestations^24^ and disease severity^25^ in previous studies, we also stratified patients by age. The DM incidence rate of UC was consistently higher than that of the control subjects in respective age groups.

A total of 13 cases of incidental PM were identified during the follow-up period, 3 in the UC group and 10 in the control subjects. The incidence rate of PM was 0.22 cases per 1000 person-years (95% CI = 0.04–0.63) in the UC cohort, close to that in the control group (0.15 cases per 1000 person-years; 95% CI = 0.07–0.28) (Table 2).

### DM and PM Incidence Rate in the Male Cohort

To clarify the relationship of sex in regards to the incidence rate of DM and PM, we stratified the patients based on sex. The incidence rate of DM was 0.13 cases per 1000 person-years (95% CI = 0.00–0.74) in the male UC cohort, compared to 0.03 cases per 1000 person-years (95% CI = 0.00–0.16) in the male control group (Table 3). The overall incidence rate of DM in the male UC cohort was consistently higher than that in the male control group.

For PM, the incidence rate of PM was 0.13 cases per 1000 person-years (95% CI = 0.00–0.74) in the male UC cohort, compared to 0.06 cases per 1000 person-years (95% CI = 0.01–0.21) in the male control group (Table 3).

### DM and PM Incidence Rate in the Female Cohort

The incidence rate of DM was 0.64 cases per 1000 person-years (95% CI = 0.17–1.63) in the female UC cohort, compared to 0.19 cases per 1000 person-years (95% CI = 0.07–0.41) in the female control group (Table 4). The incidence rate of DM in the female UC cohort was also consistently higher than that in the control group. For PM, the incidence rate of PM was 0.32 cases per 1000 person-years (95% CI = 0.04–1.14) in the female UC cohort, compared to 0.25 cases per 1000 person-years (95% CI = 0.11–0.50) in the female control group (Table 4). The overall incidence rate of PM in the female UC cohort approached to that in the female control group.

### Cumulative Incidences of Dermatomyositis and Polymyositis

**Figure 1:**
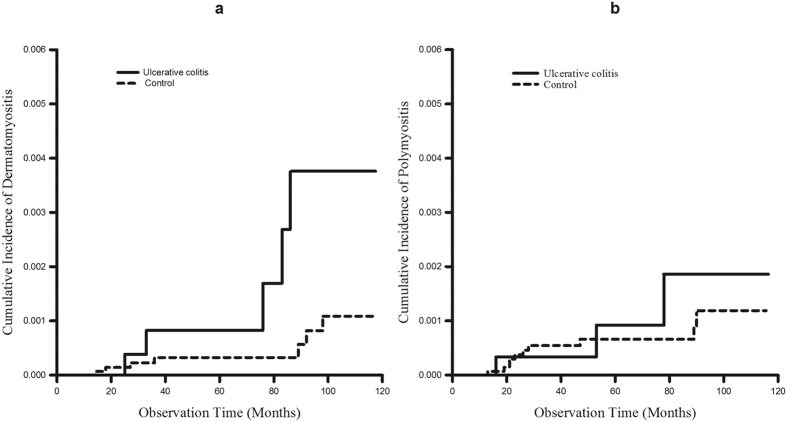
Cumulative incidence of DM and PM. **(a)** The cumulative incidence of DM was higher in UC patients than in control subjects (p = 0.026, estimated by the log-rank test). **(b)** The cumulative incidence of PM was comparable between UC patients and control subjects (p = 0.596, estimated by the log-rank test).

The cumulative incidence of DM in the UC cohort was statistically higher than that in the control group (p = 0.026; Fig. 1a) while the cumulative incidence of PM in the UC cohort was comparable to that in the control group (p = 0.596; Fig. 1b). We next performed a stratified analysis according to sex. A recurring trend of increased cumulative incidence of DM in UC patients across males and females was observed. In the male cohort, the cumulative incidence of DM in UC was higher than that in the control group, but the difference was not statistically significant (p = 0.245; Supplementary Fig. 1). With regard to PM, the cumulative incidence of PM in UC was comparable to that in the control group (p = 0.484; Supplementary Fig. 1). In the female cohort, the cumulative incidence of DM in UC was statistically higher than that in the control group (p = 0.044; Supplementary Fig. 2), while the cumulative incidence of PM in UC was comparable to that in the control group (p = 0.784; Supplementary Fig. 2).

### Hazard Ratio of Ulcerative colitis and Other Covariates in Multivariable Analyses

To determine if UC was an independent risk factor for DM, we used a multivariate Cox proportional hazard regression to calculate the hazard ratio (HR) of UC and other covariates. UC (HR: 6.19, 95% CI = 1.77–21.59, p = 0.004) in addition to RA (HR: 6.30, 95% CI = 1.71–23.22, p = 0.006), SLE (HR: 13.76, 95% CI = 2.96–64.10, p = 0.001), and SSc (HR: 6.45, 95% CI = 1.32–31.59, p = 0.021) was associated with a higher incident DM after adjusting for sex and age, RA, SLE, SSc, and UC (Table 5) concomitantly and was statistically significant.

## Discussion

In this study, the cumulative incidence of DM appears to be significantly higher in UC patients when compared with the control group, and UC can potentially be a risk factor for the co-occurrence of DM independent of other autoimmune diseases. In contrast, cumulative incidence of PM was comparable between UC patients and the control group. To the best of our knowledge, this is the first nationwide study suggesting a positive correlation of UC with incident DM. The findings of the present study support the speculation that UC could be associated with increased cumulative incidence of DM, and it leads to several interesting points of discussion.

In this study, the presence of an existing diagnosis of UC as an independent predictor of a future diagnosis of DM when compared to the control group, were consistent across sexes (0.13 vs 0.03 in men, 0.64 vs 0.19 in women) and age (0.33 vs 0.10 in young patients, 0.59 vs 0.14 in old patients) (Table 2). In previous studies, there were reports of an association between UC and DM^7^,^8^,^26^. Although the evidence is limited, these observations provide a rationale for the increased cumulative incidence of DM in UC patients as shown.

UC continues to be a major worldwide health problem, accounting for substantial costs to the health care system and society^27^. Several studies have identified several indicators that confer a poor prognosis associated with UC. Examples include: the presence of antinuclear antibodies in DM patients^28^ which are associated with uveitis, steroid dependency^29^, and paradoxical rheumatological manifestations resulting from treatment involving anti- tumour necrosis factor agents^30^. Additionally, the presence of mucocutaneous manifestations occurring in DM were also associated with primary sclerosing cholangitis in UC patients^31^. Moreover, the presence of any autoimmune disorders have also been associated with adverse outcomes of ileal pouch-anal anastomosis^32^, increased pancolitis^33^,^34^, and overall increased clinical severity^9^,^35^ in UC patients. These observations give an excellent rationale for future studies to clarify whether UC patients with DM have a more complicated clinical course than those without.

Furthermore, patients with UC have increased complications, lower remission rate of other autoimmune diseases^36^,^37^, increased recurrence rate of other autoimmune diseases^38^, and higher incidental colorectal cancer rate^39^ than those without. These findings support the need for further study of DM in patients with UC, its relationship, and its impact.

The mechanisms by which UC increases susceptibility to DM but not PM are yet to be completely elucidated. Past studies observed that DM and PM are characterized with different immunohistological features^40^, cytokine expressions^41^, autoantibodies^42^, clinical course, and prognostic factors^43^. DM and PM also showed discrepant associations with cancer subtypes in previous epidemiology studies^44^. Additionally, common genetic factors including *IRF5* rs4728142 polymorphism and *VDR* rs2228570 polymorphism are correlated with both the diagnosis of UC^3^,^4^ and DM^5^,^6^. However. there is no compelling evidence indicating that these single nucleotide polymorphisms increase PM. Thus similar gene polymorphisms in both UC and DM might explain the increased cumulative incidence of DM but not PM occurring in UC patients.

Our study has several strengths. First, we used the NHI dataset, which is a representative national sample, minimizing selection bias resulting from non-response or loss to follow-up of study subjects. In addition, the diagnoses of UC, DM, and PM were confirmed by their inclusion in the Registry of Critical Illness. However, this study also had some limitations. First, administrative databases do not contain information on statin prescription and ultraviolet exposure, which are associated with an increased DM^45^,^46^. However, previous studies suggest that statin use in UC patients is comparable to that in the general population^47^, and there is no association between ultraviolet exposure and UC incidence^48^. Therefore, the lack of information on these variables is unlikely to have introduced substantial bias. Next, this study did not examine effects of a family history of DM. To date, there have been no studies documenting associations between a family history of DM and the cumulative incidence of DM, although it is generally believed that a family history of DM implies a higher cumulative incidence of DM. However, it supported the link between UC and DM rather than refuted it even when confoundings by a family history of DM occurred.

Moreover, our database does not contain laboratory data and clinical information, such as disease phenotypes and serology reports. Thus we didn’t investigate the risk factors for the co-occurrence of UC and DM from this study. This would have been of interest to determine whether specific types of autoantibodies or disease phenotypes could be predictive of UC and DM co-occurrence. Future studies are clearly needed to address this question.

Other limitations include the relatively small number of DM cases, even in a nationwide cohort study, which might make the statistical models unstable. Regardless, this is the first nationwide study to assess the cumulative incidence of DM in UC patients and to suggest an effect of UC on cumulative incidence of DM independent of sex, age, and concomitant autoimmune diseases.

Another concern is UC patients might initiate earlier consultations with healthcare professionals for DM, because of their frequent clinic visits. However, previous studies showed little diagnosis delay in DM^49^. Furthermore, these scenarios may have affected the true cumulative incidence of DM to a certain degree, but they cannot bias the results considerably because of the short diagnosis delay in DM^49^,^50^ in addition to easy accessibility and high coverage of universal health insurance in Taiwan^13^. Lastly, we cannot rule out the possibility of miscoding in an established database that relies on physician-reported diagnoses. However, such miscoding and misclassification are likely to affect both UC and control cohorts equally and even if misclassification did result in underestimation of the association, our results still support the association of UC with DM.

In summary, this is the first preliminary observation of increased cumulative incidence of DM in patients with UC in a nationwide cohort study. Our study raises the possibility that co-occurrence of common as well as rare autoimmune diseases could help elucidate the pathophysiologic background of rare autoimmune diseases that are not addressed extensively due to their low prevalence. In addition, genetic components (*IRF5* rs4728142 and *VDR* rs2228570) common to UC and DM suggest that a common treatment may be effective against both these diseases. Current treatments for UC probably become novel treatment options for DM, for which it has been difficult to develop specific treatment due to its low frequency. For example, tacrolimus has been shown to exert therapeutic effects similar to biologics in UC^51^. There are also reports demonstrating its efficacy in DM^52^. Moreover, co-existing UC and DM might represent unique entities different from either process alone. Further research is necessary to confirm our findings, to clarify the clinical course of UC associated with DM, and to determine whether these patients require unique therapeutic approaches to achieve remission of either disease.

## Additional Information

**How to cite this article**: Tseng, C.-C. *et al*. Increased Cumulative Incidence of Dermatomyositis in Ulcerative Colitis: a Nationwide Cohort Study. *Sci. Rep.* **6**, 28175; doi: 10.1038/srep28175 (2016).

## Supplementary Material

Supplementary Information

## Figures and Tables

**Table 1:** The age distribution of patients with ulcerative colitis and control subjects in baseline.

	Males		Females	
Gender	UC	Control subjects	UC	Control subjects
Number	(n = 1704)	(n = 7955)	(n = 1429)	(n = 6771)
Age(years; mean ± SD)	50.42 ± 16.52	49.70 ± 16.00	53.76 ± 15.38	53.02 ± 14.82
Age group	n (%)	n (%)	n (%)	n (%)
> = 20, <30	202 (11.85)	948 (11.92)	89 (6.23)	419 (6.19)
> = 30, <40	303 (17.78)	1438 (18.08)	196 (13.72)	937 (13.84)
> = 40, <50	351 (20.60)	1732 (21.77)	295 (20.64)	1471 (21.73)
> = 50, <60	320 (18.78)	1527 (19.20)	318 (22.25)	1593 (23.53)
> = 60, <70	265 (15.55)	1260 (15.84)	271 (18.96)	1279 (18.89)
> = 70, <80	205 (12.03)	850 (10.69)	203 (14.21)	884 (13.06)
> = 80, <90	55 (3.23)	194 (2.44)	54 (3.78)	182 (2.69)
> = 90	3 (0.18)	6 (0.08)	3 (0.21)	6 (0.09)
p	0.298		0.244	

SD: standard deviation.

**Table 2:** Incidence rate of dermatomyositis and polymyositis in ulcerative colitis patients and control subjects stratified by age.

Age at baseline (years)	UC (n = 3133)			Control subjects (n = 14726)				
	No.^a^	follow-up^b^	Incidence rate^c^	95% CI	No.	follow-up	Incidence rate	95% CI
DM								
20–69	4	12101.58	0.33	0.09–0.85	6	58502.42	0.10	0.04–0.22
> = 70	1	1699.92	0.59	0.01–3.28	1	7006.42	0.14	0.00–0.79
Total	5	13801.50	0.36	0.12–0.85	7	65508.83	0.11	0.04–0.22
PM								
Total	3	13822.83	0.22	0.04–0.63	10	65481.17	0.15	0.07–0.28

^a^Numbers of new diagnosed DM/PM.

^b^The total follow-up period (person-years).

^c^Incidence rate per 1000 person-years.

**Table 3:** Incidence rate of dermatomyositis and polymyositis in men.

Age at baseline (years)	UC (n = 1704)			Control subjects (n = 7955)				
	No.^a^	follow-up^b^	Incidence rate^c^	95% CI	No.	follow-up	Incidence rate	95% CI
DM								
Total	1	7506.17	0.13	0.00–0.74	1	33785.25	0.03	0.00–0.16
PM								
Total	1	7507.83	0.13	0.00–0.74	2	33784.67	0.06	0.01–0.21

^a^Numbers of new diagnosed DM/PM.

^b^The total follow-up period (person-years).

^c^Incidence rate per 1000 person-years.

**Table 4:** Incidence rate of dermatomyositis and polymyositis in women.

Age at baseline (years)	UC (n = 1429)			Control subjects (n = 6771)				
	No.^a^	follow-up^b^	Incidence rate^c^	95% CI	No.	follow-up	Incidence rate	95% CI
DM								
Total	4	6295.33	0.64	0.17–1.63	6	31723.58	0.19	0.07–0.41
PM								
Total	2	6315.00	0.32	0.04–1.14	8	31696.50	0.25	0.11–0.50

^a^Numbers of new diagnosed DM/PM.

^b^The total follow-up period (person-years).

^c^Incidence rate per 1000 person-years.

**Table 5:** The hazard ratio of various autoimmune diseases for dermatomyositis in multivariable analyses.

Variables	HR^a^ (95% CI)	p
UC	6.19 (1.77–21.59)	0.004
RA	6.30 (1.71–23.22)	0.006
SLE	13.76 (2.96–64.10)	0.001
SSc	6.45 (1.32–31.59)	0.021

^a^Hazard ratio, adjusted for age, sex, UC, RA, SLE, and SSc.

## References

[b1] Molodecky N. A. . Increasing incidence and prevalence of the inflammatory bowel diseases with time, based on systematic review. Gastroenterology 142, 46–54 (2012). 22001864 10.1053/j.gastro.2011.10.001

[b2] Wu H. & Shen B. Pouchitis: lessons for inflammatory bowel disease. Curr Opin Gastroenterol 25, 314–322 (2009). 19349860 10.1097/MOG.0b013e32832b36eb

[b3] Xue L. N. . Associations between vitamin D receptor polymorphisms and susceptibility to ulcerative colitis and Crohn’s disease: a meta-analysis. Inflamm Bowel Dis. 19, 54–60 (2013). 22467262 10.1002/ibd.22966

[b4] Jostins L. . Host-microbe interactions have shaped the genetic architecture of inflammatory bowel disease. Nature 491, 119–124 (2012). 23128233 10.1038/nature11582 PMC3491803

[b5] Dzhebir G. . Association of vitamin D receptor gene BsmI B/b and FokI F/f polymorphisms with adult dermatomyositis and systemic lupus erythematosus. Int J Dermatol , doi: 10.1111/ijd.13263 (2016). 26972080

[b6] Chen S. . Genetic association study of TNFAIP3, IFIH1, IRF5 polymorphisms with polymyositis/dermatomyositis in Chinese Han population. PLoS One 9, e110044 (2014). 25337792 10.1371/journal.pone.0110044 PMC4206287

[b7] Ray D. W. . Transverse myelitis as the presentation of Jo-1 antibody syndrome (myositis and fibrosing alveolitis) in long-standing ulcerative colitis. Br J Rheumatol. 32, 1105–1108 (1993). 8252324 10.1093/rheumatology/32.12.1105

[b8] Bodoki L., Nagy-Vincze M., Griger Z., Péter A. & Dankó K. Anti-NXP2-positive dermatomyositis associated with ulcerative colitis and celiac disease. Orv Hetil. 155, 1033–1038 (2014). 24954145 10.1556/OH.2014.29940

[b9] Ravi K. . Inflammatory bowel disease in the setting of autoimmune pancreatitis. Inflamm Bowel Dis. 15, 1326–1330 (2009). 19235915 10.1002/ibd.20898

[b10] Naji P., Shahram F., Nadji A. & Davatchi F. Effect of early treatment in polymyositis and dermatomyositis. Neurol India 58, 58–61 (2010). 20228465 10.4103/0028-3886.60398

[b11] Hilton-Jones D. Inflammatory muscle diseases. Curr Opin Neurol. 14, 591–596 (2001). 11562570 10.1097/00019052-200110000-00007

[b12] Page G., Chevrel G. & Miossec P. Anatomic localization of immature and mature dendritic cell subsets in dermatomyositis and polymyositis: Interaction with chemokines and Th1 cytokine-producing cells. Arthritis Rheum. 50, 199–208 (2004). 14730617 10.1002/art.11428

[b13] *Bureau of National Health Insurance. Universal Health Coverage in Taiwan.* Available at: http://www.nhi.gov.tw/Resource/webdata/21717_1_20120808UniversalHealthCoverage.pdf (Accessed: 15th December 2015).

[b14] Yang N. P. . Epidemiological survey of orthopedic joint dislocations based on nationwide insurance data in Taiwan, 2000–2005. BMC Musculoskelet Disord. 12, 253 (2011). 22053727 10.1186/1471-2474-12-253 PMC3228707

[b15] Bohan A. & Peter J. B. Polymyositis and dermatomyositis (first of two parts). N Engl J Med. 292, 344–347 (1975). 1090839 10.1056/NEJM197502132920706

[b16] Bohan A. & Peter J. B. Polymyositis and dermatomyositis (second of two parts). N Engl J Med. 292, 403–407 (1975). 1089199 10.1056/NEJM197502202920807

[b17] Chuang C. H. . Increasing incidence and lifetime risk of inflammatory bowel disease in Taiwan: a nationwide study in a low-endemic area 1998–2010. Inflamm Bowel Dis. 19, 2815–2819 (2013). 24141711 10.1097/01.MIB.0000435436.99612.27

[b18] Chen S. J. . Prevalence of autoimmune diseases in in-patients with schizophrenia: nationwide population-based study. Br J Psychiatry 200, 374–380 (2012). 22442099 10.1192/bjp.bp.111.092098

[b19] Kuo C. F. . Familial Aggregation of Systemic Lupus Erythematosus and Coaggregation of Autoimmune Diseases in Affected Families. JAMA Intern Med. 175, 1518–1526 (2015). 26193127 10.1001/jamainternmed.2015.3528

[b20] Lai Y. T. . Dermatomyositis is associated with an increased risk of cardiovascular and cerebrovascular events: a Taiwanese population-based longitudinal follow-up study. Br J Dermatol. 168, 1054–1059 (2013). 23330740 10.1111/bjd.12245

[b21] Wu C. S., Gau S. S. & Lai M. S. Long-term antidepressant use and the risk of type 2 diabetes mellitus: a population-based, nested case-control study in Taiwan. J Clin Psychiatry 75, 31–38 (2014). 24502860 10.4088/JCP.13m08421

[b22] Aguila L. A. . Clinical and laboratory features of overlap syndromes of idiopathic inflammatory myopathies associated with systemic lupus erythematosus, systemic sclerosis, or rheumatoid arthritis. Clin Rheumatol. 33, 1093–1098 (2014). 24989017 10.1007/s10067-014-2730-z

[b23] Bernard R. In Fundamentals of Biostatistics. seventh edn , Ch. 14, 725–810 (Cengage Learning, 2010).

[b24] Vegh Z. . Association of extraintestinal manifestations and anaemia with disease outcomes in patients with inflammatory bowel disease. Scand J Gastroenterol 51, 848–854 (2016). 26880133 10.3109/00365521.2016.1140807

[b25] Reenaers C., Pirard C., Vankemseke C., Latour P., Belaiche J. & Louis E. Long-term evolution and predictive factors of mild inflammatory bowel disease. Scand J Gastroenterol 51, 712–719 (2016). 26815198 10.3109/00365521.2015.1128965

[b26] Oshitani H., Wakabayashi Y., Sawaguchi Y., Koike H. & Yoshino Y. A case of dermatomyositis followed by multiple mononeuritis, with the history of ulcerative colitis and Basedow’s disease. J Kyorin Med Soc . 12, 47–53 (1981).

[b27] Burisch J., Jess T., Martinato M. & Lakatos P. L. ECCO -EpiCom. The burden of inflammatory bowel disease in Europe. J Crohns Colitis . 7, 322–337 (2013). 23395397 10.1016/j.crohns.2013.01.010

[b28] Koler R. A. & Montemarano A. Dermatomyositis. Am Fam Physician 64, 1565–1572 (2001). 11730311

[b29] Barahona-Garrido J. . Antinuclear antibodies: a marker associated with steroid dependence in patients with ulcerative colitis. Inflamm Bowel Dis. 15, 1039–1043 (2009). 19107779 10.1002/ibd.20852

[b30] Coutzac C. . Association Between Infliximab Trough Levels and the Occurrence of Paradoxical Manifestations in Patients with Inflammatory Bowel Disease: a Case-Control Study. J Crohns Colitis . 9, 982–987 (2015). 26351388 10.1093/ecco-jcc/jjv159

[b31] Khosravi Khorashad A. . Frequency and risk factors of primary sclerosing cholangitis among patients with inflammatory bowel disease in North-East of Iran. Gastroenterol Hepatol Bed Bench . 8, 200–206 (2015). 26328042 PMC4553160

[b32] Shen B. . Association between immune-associated disorders and adverse outcomes of ileal pouch-anal anastomosis. Am J Gastroenterol 104, 655–664 (2009). 19262522 10.1038/ajg.2008.76

[b33] Ordonez F. . Pediatric ulcerative colitis associated with autoimmune diseases: a distinct form of inflammatory bowel disease? Inflamm Bowel Dis. 18, 1809–1817 (2012). 22238154 10.1002/ibd.22864

[b34] Oxford E. C. . Impact of coexistent celiac disease on phenotype and natural history of inflammatory bowel diseases. Am J Gastroenterol 108, 1123–1129 (2013). 23419379 10.1038/ajg.2013.20 PMC3845216

[b35] Rönnblom A., Holmström T., Tanghöj H., Wanders A. & Sjöberg D. Celiac disease, collagenous sprue and microscopic colitis in IBD. Observations from a population-based cohort of IBD (ICURE). Scand J Gastroenterol 50, 1234–1240 (2015). 25921772 10.3109/00365521.2015.1041152

[b36] Perdigoto R., Carpenter H. A. & Czaja A. J. Frequency and significance of chronic ulcerative colitis in severe corticosteroid-treated autoimmune hepatitis. J Hepatol. 14, 325–331 (1992). 1500696 10.1016/0168-8278(92)90178-r

[b37] Bailey J. . Autoimmune hepatitis with inflammatory bowel disease is distinct and may be more refractory to traditional treatment. Am J Gastroenterol 109, S149 (2014).

[b38] Ravikumar R. . Risk factors for recurrent primary sclerosing cholangitis after liver transplantation. J Hepatol. 63, 1139–1146 (2015). 26186988 10.1016/j.jhep.2015.07.005

[b39] Vera A. . Colorectal cancer in patients with inflammatory bowel disease after liver transplantation for primary sclerosing cholangitis. Transplantation 75, 1983–1988 (2003). 12829898 10.1097/01.TP.0000058744.34965.38

[b40] Choi J. H. . Differential immunohistological features of inflammatory myopathies and dysferlinopathy. J Korean Med Sci. 24, 1015–1023 (2009). 19949654 10.3346/jkms.2009.24.6.1015 PMC2775846

[b41] Aleksza M. . Altered cytokine expression of peripheral blood lymphocytes in polymyositis and dermatomyositis. Ann Rheum Dis. 64, 1485–1489 (2005). 15829578 10.1136/ard.2003.017715 PMC1755238

[b42] Petri M. H. . Implications in the difference of anti-Mi-2 and -p155/140 autoantibody prevalence in two dermatomyositis cohorts from Mexico City and Guadalajara. Arthritis Res Ther. 15, R48 (2013). 23557279 10.1186/ar4207 PMC4060281

[b43] Galindo-Feria A. S., Rojas-Serrano J. & Hinojosa-Azaola A. Clinical and Prognostic Factors Associated With Survival in Mexican Patients With Idiopathic Inflammatory Myopathies. J Clin Rheumatol . 22, 51–56 (2016). 26906295 10.1097/RHU.0000000000000365

[b44] Hill C. L. . Frequency of specific cancer types in dermatomyositis and polymyositis: a population-based study. Lancet 357, 96–100 (2001). 11197446 10.1016/S0140-6736(00)03540-6

[b45] Sailler L. . Increased exposure to statins in patients developing chronic muscle diseases: a 2-year retrospective study. Ann Rheum Dis. 67, 614–619 (2008). 17768174 10.1136/ard.2007.075523

[b46] Okada S. . Global surface ultraviolet radiation intensity may modulate the clinical and immunologic expression of autoimmune muscle disease. Arthritis Rheum. 48, 2285–2293 (2003). 12905483 10.1002/art.11090

[b47] Dregan A., Charlton J., Chowienczyk P. & Gulliford M. C. Chronic inflammatory disorders and risk of type 2 diabetes mellitus, coronary heart disease, and stroke: a population-based cohort study. Circulation. 130, 837–844 (2014). 24970784 10.1161/CIRCULATIONAHA.114.009990

[b48] Nerich V. . Low exposure to sunlight is a risk factor for Crohn’s disease. Aliment Pharmacol Ther 33, 940–945 (2011). 21332762 10.1111/j.1365-2036.2011.04601.x

[b49] Holden D. J., Brownell A. K. & Fritzler M. J. Clinical and serologic features of patients with polymyositis or dermatomyositis. Can Med Assoc J. 132, 649–653 (1985). 3872156 PMC1345785

[b50] Airio A., Kautiainen H. & Hakala M. Prognosis and mortality of polymyositis and dermatomyositis patients. Clin Rheumatol. 25, 234–239 (2006). 16477398 10.1007/s10067-005-1164-z

[b51] Yamamoto T., Shimoyama T., Umegae S. & Matsumoto K. Tacrolimus vs. anti-tumour necrosis factor agents for moderately to severely active ulcerative colitis: a retrospective observational study. Aliment Pharmacol Ther. 43, 705–716 (2016). 26762838 10.1111/apt.13531

[b52] Yokoyama Y., Furuta S., Ikeda K., Hirose K. & Nakajima H. Corticosteroid-sparing effect of tacrolimus in the initial treatment of dermatomyositis and polymyositis. Mod Rheumatol . 25, 888–892 (2015). 25775144 10.3109/14397595.2015.1029239

