# Supplementary material for: Increased Cumulative Incidence of Dermatomyositis in Ulcerative Colitis: a Nationwide Cohort Study
Source: Sci Rep. 2016 Jun 21;6:28175. doi: 10.1038/srep28175 (PMC4914943; doi:10.1038/srep28175)
Supplement: Supplementary Information [file srep28175-s1.pdf]

Increased Cumulative Incidence of Dermatomyositis in Ulcerative Colitis: a Nationwide Cohort Study

Chia-Chun Tseng[1,2], Shun-Jen Chang[3,*], Wei-Ting Liao[4], Ya-Ting Chan[3], Wen-Chan Tsai[5], Tsan-Teng Ou[5], Cheng-Chin Wu[5], Wan-Yu Sung[5,6], Ming-Chia Hsieh[7,8], Jeng-Hsien Yen[5,6,9,*]

[1]Graduate Institute of Clinical Medicine, College of Medicine, Kaohsiung Medical University, Kaohsiung, Taiwan

[2]Department of Internal Medicine, Kaohsiung Municipal Ta-Tung Hospital, Kaohsiung, Taiwan

[3]Department of Kinesiology, Health and Leisure Studies, National University of Kaohsiung, Kaohsiung, Taiwan

[4]Department of Biotechnology, College of Life Science, Kaohsiung Medical University, Kaohsiung, Taiwan

[5]Division of Rheumatology, Department of Internal Medicine, Kaohsiung Medical University Hospital, Kaohsiung, Taiwan

[6]Graduate Institute of Medicine, College of Medicine, Kaohsiung Medical University, Kaohsiung, Taiwan

[7]Division of Endocrinology and Metabolism, Department of Internal Medicine, Changhua Christian Hospital, Changhua, Taiwan

[8]Graduate Institute of Integrated Medicine, China Medical University, Taichung, Taiwan

[9]Institute of Biomedical Sciences, National Sun Yat-sen University, Kaohsiung, Taiwan

[*]Correspondence and requests for materials should be addressed to S.-J.C. (email: changsj1104@gmail.com) or J.-H.Y. (email: jehsye@kmu.edu.tw).

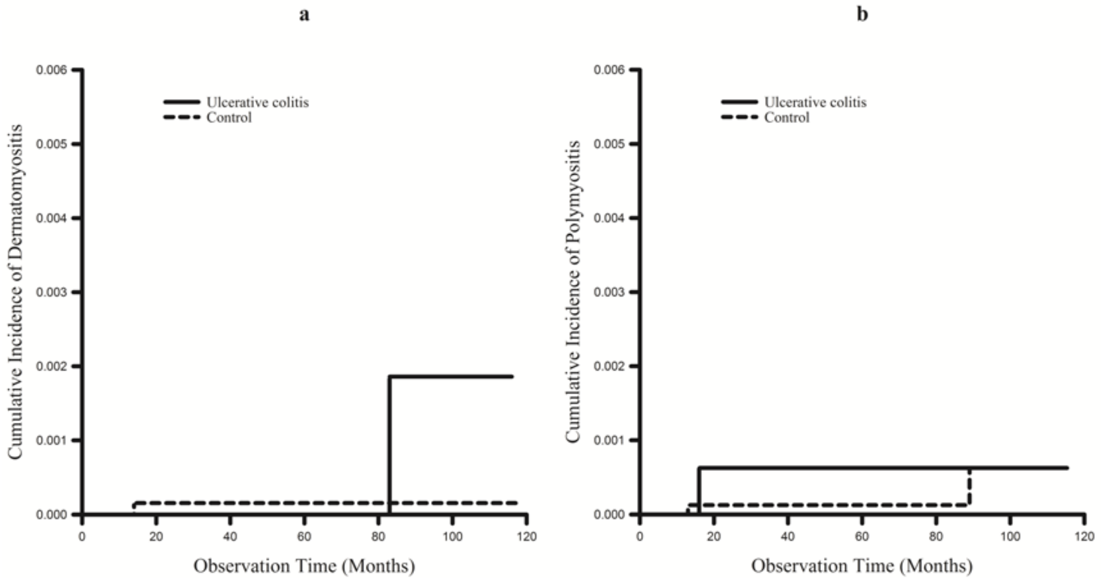

Supplementary Figure 1. Cumulative incidence of DM and PM in men. **(a)**The cumulative incidence of DM in men was higher in UC patients than in control subjects, but the difference was not statistically significant (p=0.245, estimated by the log-rank test). **(b)**The cumulative incidence of PM in men was comparable between UC patients and control subjects (p=0.484, estimated by the log-rank test).

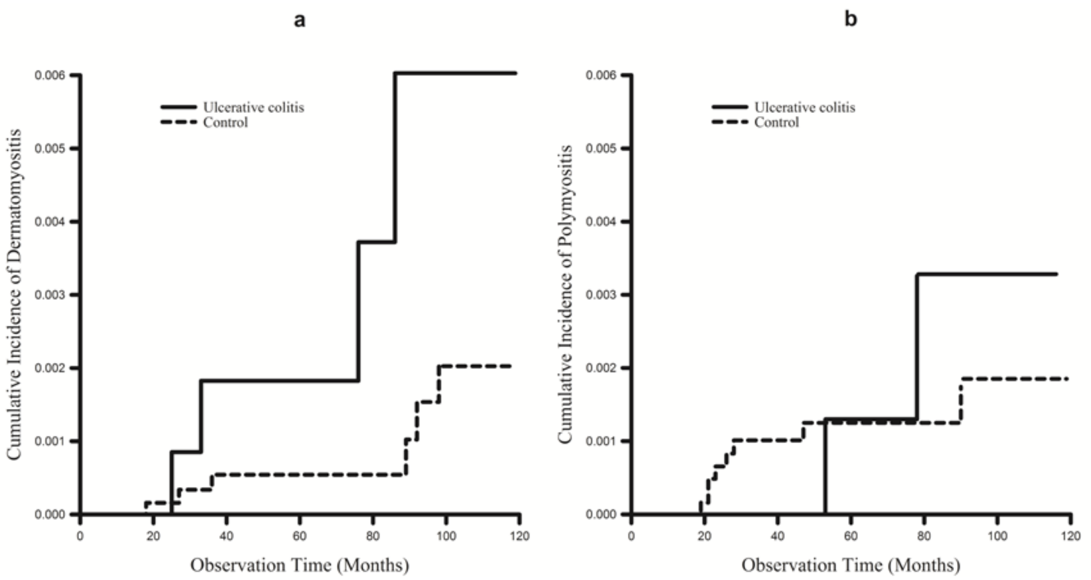

Supplementary Figure 2. Cumulative incidence of DM and PM in women. **(a)**The cumulative incidence of DM in women was higher in UC patients than in control subjects (p=0.044, estimated by the log-rank test). **(b)**The cumulative incidence of PM in women was comparable between UC patients and control subjects (p=0.784, estimated by the log-rank test).
